# Mental health after a critical incident: the role of parental status and prior training in firefighters

**DOI:** 10.3389/fpsyt.2026.1771437

**Published:** 2026-04-13

**Authors:** Vanessa Borck, Tabea Görlich, Francesco Pahnke, Tristan Wellendorff, Finja Marten, Ulrich Wesemann

**Affiliations:** 1 Department of Psychiatry, Psychotherapy and Psychotraumatology, Bundeswehrkrankenhaus Berlin, Berlin, Germany; 2 Klinik für Psychiatrie und Psychotherapie, Charité - Universitätsmedizin Berlin, Berlin, Germany

**Keywords:** accident, anger, child, firefighters, mental health, PTSD, quality of life, sleep

## Abstract

**Introduction:**

In this brief research report, we postulated that operations involving children can increase stress for first responders. Here, a traffic accident in which a mother and her child were fatally wounded was investigated. We postulated that firefighters with children and firefighters without pre-training experience would experience greater psychological impact when responding to this incident.

**Methods:**

All firefighters who responded to the incident could participate voluntarily. Participants (*n* = 49) were categorized by parental status (*n* = 24 parents; *n* = 25 non-parents) and pre-deployment training (yes: *n* = 25; no: *n* = 22) and analyzed using a factorial design including deployment exposure. The design permitted examination of family-related factors and preparatory training effects. Alongside sociodemographic data, the following data were collected: quality of life (WHOQOL-BREF), anger (STAXI-2), post-traumatic stress disorder (PCL-5) and daytime sleepiness (ESS).

**Results:**

Firefighters with children reported lower environmental quality of life (F(1,45) = 4.27, *p* = .045) and physical quality of life (F(1,45) = 4.11, *p* = .049). Significant interaction effects between parental status and deployment exposure were observed for temperament (F(1,44) = 5.83, *p* = .020). Pre-deployment training was associated with lower temperament (F(1,40) = 9.26, *p* = .004) and anger expression-out (F(1,41) = 11.99, *p* = .001). Deployment exposure alone showed no independent effects.

**Conclusion:**

The psychological consequences of critical events appear to be influenced more by individual characteristics and preparation than by exposure alone. Targeted preparatory training can reduce anger-related reactions, particularly among firefighters directly involved in operations.

## 1 Introduction

Firefighters are regularly exposed to potentially traumatic events (1). These include the rescue and care of critically injured individuals, as well as working under physically and psychologically demanding conditions (2–4). Furthermore, firefighters are also involved in so-called major incidents, also known as critical incidents, defined by a large number of casualties and injured parties (5). Examples of major incidents include deliberately caused events, such as terrorist attacks or rampages, as well as accidents involving multiple fatalities or assignments concerning killed or wounded children. Such deployments are regarded as being of a particularly challenging nature, as they have been demonstrated to result in elevated levels of psychological distress among emergency personnel (6). Recent evidence indicates a high prevalence of exposure to critical incidents in firefighters. For instance, MacDermid et al. reported that 85% of firefighters surveyed had been deployed to at least one critical incident within a two-month period (7).

With the general rise in deployment numbers, an increase in the frequency of critical incidents may be expected. As operational demands grow, so does the statistical likelihood of being involved in such incidents, which could lead to greater cumulative exposure and increased psychological strain. When comparing the years 2013 (379,521 operations) and 2023 (514,866 operations), the Berlin Fire Department saw an increase of around 36% in the total number of deployments (8, 9). It is to be expected that, in view of the rising deployment numbers, there will be an increase in workload in fire departments. Sandrin et al. showed that higher workload is associated with greater perceived stress and poorer perceived health and performance among firefighters (10). However, the rise of total deployments must also be viewed in the context of larger trends such as the simultaneous growth of the population of Berlin and should therefore not be interpreted as a linear correlation (11).

In addition to the increasing number of operations, organizational working conditions are also described as relevant stressors in emergency service personnel. Working conditions that have a physical and mental strain include shift work, weekend work and the accumulation of overtime hours (12). Studies among professional firefighters have also reported high rates of sleep deprivation, depressive symptoms, and reduced well-being, all of which are associated with psychosomatic strain (13). After a terrorist attack in Berlin, firefighters were found to have reduced environmental and physical quality of life, which was evident not only immediately after the event, but also two years later (14, 15). The psychological burden can also be seen in the prevalence of symptoms of post-traumatic stress disorder (PTSD). Berger et al. showed a current PTSD prevalence of 10% among rescue workers, with “current” defined as meeting diagnostic criteria within the past 1–6 months (16). A meta-analysis conducted 12 years later by Martínez et al. confirmed these findings by yielding comparable results (17). In contrast, studies of the general population report substantially lower rates, with between 1.4% meeting PTSD criteria within the past month and 2.8% within the past year (18). Despite the non-identical reference periods, the observed discrepancy indicates a substantially elevated PTSD burden among rescue workers in comparison to the general population.

To counteract the increased psychological burden, various pre- and post-incident interventions have been implemented by Berlin Fire Department, which are intended to prevent mental illness and incapacity for duty. A central component of these preventive strategies is training drills focused on the deployment in hazardous situations and the care for multiple patients in the event of major incidents. In addition, tactical medical care for injured civilians/people in the event of a mass casualty incident is also part of the regular training schedule (19). The training and continuing education that firefighters complete as part of their professional duties are also part of various pre- and post-incident interventions. Additionally, there are individual coping strategies including the personal characteristics of the emergency personnel, which play a key role in processing stressful operations. These include factors such as individual resilience, emotional stability as well as social support in the private environment and the individual life situation (20).

Despite preparation and coping strategies, certain assignments, for example involving children, can be particularly challenging. In their review, Fraess-Phillips et al. reported that the highest levels of stress among firefighters are observed following traumatic events involving children, particularly when personal identification processes are involved (21). The fact that the death of a child during an operation is also a particularly great emotional and psychological challenge was shown by Del Ben et al. (22). In this study, an analysis of firefighters’ self-identified “single worst event” revealed that 34% of respondents who answered the question reported that the death of a child was the most distressing experience of their professional career.

In addition, the type of serious incident, the frequency, individual coping strategies, and the services offered by individual organizations to prepare for deployment and debrief after deployment were identified as important factors affecting mental health (23). As well as direct exposure, emergency personnel who were not deployed to the scene may also experience psychological responses to critical incidents. Information about severe incidents can spread rapidly within emergency organizations through operational reports, internal communication or media coverage, which can lead to indirect psychological stress. These processes are related to the concept of secondary traumatic stress, which is defined as a psychological reaction resulting from exposure to the suffering or traumatic experiences of others rather than from direct personal involvement (24). Previous research has shown that indirect exposure to traumatic events can lead to post-traumatic stress symptoms and emotional distress among first responders (25). Consequently, both directly deployed and non-deployed firefighters may experience psychological reactions following highly distressing incidents within their organization.

In March 2024, a fatal traffic accident occurred in the center of Berlin, resulting in the death of a mother and her four-year-old child. Five other people were injured. Forty-eight members of the Berlin Fire Department were involved in the ensuing operation (26). As already mentioned, incidents involving children are described in the literature as being particularly distressing for emergency responders (4). However, few studies have examined how parental status and preparatory training may influence psychological responses to critical incidents involving children.

This study is part of the CASH (Coping Anxiety Stress Hostility) project, which investigates the psychological burden of civilian and military forces after potentially stressful major incidents. It aims to develop improved concepts for the psychosocial care of first responders before and after incidents (14). These first responders include personnel from organizations such as fire departments, police officers, rescue services and the Federal Agency for Technical Relief.

The aim of our study is to examine the psychological status of firefighters after the aforementioned traffic accident. The central questions are 1. whether there is a difference in psychological burden between parents and non-parents, and 2. whether there is a difference in psychological burden between emergency services with and without the described pre-training measures. In addition, we explored whether deployment exposure moderated these associations. We hypothesized that firefighters with children and those without pre-deployment training would report greater psychological burden following the incident.

## 2 Materials and methods

Data collection: The initial contact was made via the leadership of the Berlin Fire Department. Contact was then made with the station leaders or unit commanders to identify the service personnel involved. As a comparison group, all firefighters from the same units had the opportunity to participate in the study. The data were collected by means of a questionnaire 6 months after the traffic accident in March 2024 by employees of the Department of Psychotraumatology at the Bundeswehrkrankenhaus Berlin. Participation was voluntary, written consent was obtained for all participants. The inclusion criteria were active membership of the Berlin Fire Department and an age range of 18–65 years. All firefighters who took part in the survey were taken off duty for the duration of the survey to limit distraction. Extra rooms were made available for the completion of the questionnaire.

All participants had the opportunity to contact the Department of Psychotraumatology at the Bundeswehrkrankenhaus Berlin in the event of emotional problems in connection with the incident. The offer included in-depth counseling up to inpatient psychiatric admission. In addition, the interview was accompanied by the emergency psychosocial service (EPS) and/or the emergency psychologist of the Berlin Fire Department.

A positive ethics vote by the Ethics Committee of the Charité Berlin (protocol code: EA4/085/18) was obtained.

A total of 49 firefighters took part in the study. The sample included firefighters from both the deployed group (DG; *n* = 19) and the control group (CG; *n* = 30). Members of the control group were off duty, absent due to illness or attending other duties on the day of the incident. In total, 48 firefighters from the Berlin Fire Department were involved in the operation.

Measurement instruments: We used a “basic questionnaire” to collect sociodemographic data (age, gender, years of service, parental status). This questionnaire also asked whether participants had received training prior to deployment. If participants indicated that they were parents, they were also asked about the age of their youngest child (“*Do you have children, and if so, how old is the youngest?*”). Only children under 18 years living in the participant’s household were considered. In addition, the following validated questionnaires were utilized for the purpose of the data collection: The Posttraumatic Stress Disorder Checklist (PCL-5) was used to evaluate symptoms of post-traumatic stress according to the criteria set out in the DSM-5. This 20-item instrument uses a 5-point Likert scale (0 = “not at all” to 4 = “extremely”) to assess PTSD symptoms over the past month. It assesses intrusion, avoidance, negative alterations in mood and cognition, and hyperarousal, and provides a total symptom severity score. A cut-off score of 33 was used to indicate PTSD (27).

Quality of life was assessed using the World Health Organization Quality of Life Questionnaire (WHOQOL-BREF). It is a 26-item self-report questionnaire to measure four domains of quality of life: physical health (item 15 *“How well can you get around?”)* and psychological health (e.g., item 11 *“Can you accept your appearance?”*), social relationships (item 20 “*How satisfied are you with your personal relationships?”*) and the environment conditions (e.g., item 8 *“How safe do you feel in your daily life?”*). Each item is rated on a five-point Likert scale, with higher scores indicating better quality of life (28).

The State–Trait Anger Expression Inventory-2 (STAXI-2) was used to assess different dimensions of anger. On the one hand, situational anger (state anger) is measured, i.e., the intensity of the momentary sensation of anger. Furthermore, there is the possibility to assess different dimensions of anger. These include trait anger with the components temperament and anger reaction, the forms of anger expression (anger-expression in and anger-expression out) and anger control (29). This instrument was included because previous studies have reported elevated STAXI scores in first responders exposed to critical incidents (30). The questionnaire allows a multidimensional assessment of anger experience and regulation. In addition, the extent to which people tend to express anger openly (anger-out), suppress it (anger-in) or consciously regulate it (anger-control) is recorded.

The Epworth Sleepiness Scale (ESS) examines daytime sleepiness in recent weeks. Retrospectively, the subjective assessment of the probability of falling asleep in eight given typical situations is asked. Here, the cut-off value for increased daytime sleepiness was 11 points (31). We included this instrument because previous studies have shown that firefighters score higher on the ESS in terms of daytime sleepiness (32).

Statistical evaluation: Metric sociodemographic data from DG and CG groups were compared using the *t*-test for independent samples and the chi-square test (*×²* test) for categorical data. The point prevalences for PTSD were calculated as a percentage of participants meeting the cut-off (yes/no). Participants were categorized according to deployment exposure, parental status (parents: *n* = 24; non-parents: *n* = 25), and pre-deployment training (training: *n* = 25; no training: *n* = 22) for inclusion in the respective factorial analyses. Some participants did not complete all items, so group sizes and degrees of freedom differ depending on the degree to which the respective questionnaires were completed.

For the factorial analyses, participants were classified according to deployment exposure (deployed vs. non-deployed), parental status (parents vs. non-parents), and pre-deployment training (training vs. no training). To examine psychological outcomes as a function of parental status and deployment exposure, separate two-factor analyses of variance (ANOVAs) were conducted for each outcome variable, including parental status and deployment exposure as between-subject factors. To investigate the role of preparatory training, additional two-factor ANOVAs were performed including deployment exposure and pre-deployment training as between-subject factors. Prior to inferential analyses, assumptions of normality were assessed using Q–Q plots. Visual inspection indicated that the distributions were approximately normal. Homogeneity of variance was evaluated using Levene’s test. The significance level was set at α = .05. Partial eta squared (*ηp2*) is reported as a measure of effect size.

All statistical analyses of the data were carried out using SPSS (Statistics for Windows, version 21. Armonk, NY, USA: IBM Corp.).

## 3 Results

Descriptive statistics of age and years of service are presented in **Table 1**. The mean age of all participants was 35.7 years, with a range of 22 to 59 years. The median period of service was 9 years. Of the 49 participants, 2 were female and 47 were male. Among participants who reported being parents, the mean age of the youngest child was 3.3 years (SD = 2.36), with a median of 2 years. Overall, internal consistency was good to very good (STAXI Temperament: α = .86; Trait Anger: α = .85; Anger Expression-Out: α = .93; WHO-QoL: α = .92; ESS: α = .80).

**Table 1:** Age and service experience of firefighters in the survey.

Variable	*n*	Mean	Median	Standard deviation	Q1	Q3
Age	48	35.65	35	9.22	29	39
YoS	49	9.33	6	8.64	3.5	11.5

YoS= years of service.

### 3.1 Prevalence of PTSD

Using the recommended PCL-5 cut-off score (≥ 33), no participant met criteria for PTSD.

### 3.2 Psychological outcomes as a function of parental status and deployment exposure

Two-factor analyses of variance were used to examine psychological outcomes, including parental status (parents vs. non-parents) and deployment exposure (deployed vs. non-deployed). The results of these analyses are presented in **Table 2**. A significant main effect of parental status was observed for environmental quality of life, *F (1*,45) = 4.27, *p* = .045, indicating lower scores among firefighters with children. A similar effect was found for physical quality of life, *F (1*,45) = 4.11, *p* = .049. Significant interaction effects between parental status and deployment exposure were observed for temperament, *F (1*,44) = 5.83, *p* = .020, anger reaction, *F (1*,44) = 4.59, *p* = .038, and anger expression-out, *F (1*,45) = 4.28, *p* = .044. No other significant main effects of deployment exposure were observed across the examined psychological outcomes.

**Table 2:** Two-factor ANOVAs examining parental status and deployment exposure.

Outcome variable	Effect	*F* (df1, df2)	*p*	*η_p_²*
WHOQOL-BREF Environment	Parents	4.27 (1,45)	**.045**	.087
	Deployment	0.80 (1,45)	.375	.018
	Parents × Deployment	0.01 (1,45)	.909	.000
WHOQOL-BREF Physical	Parents	4.11 (1,45)	**.049**	.084
	Deployment	0.00 (1,45)	.963	.000
	Parents × Deployment	0.26 (1,45)	.611	.006
ESS	Parents	3.53 (1,45)	.067	.073
	Deployment	0.28 (1,45)	.598	.006
	Parents × Deployment	0.17 (1,45)	.686	.004
STAXI Anger Ex Out	Parents	1.25 (1,45)	.269	.027
	Deployment	0.38 (1,45)	.541	.008
	Parents × Deployment	4.28 (1,45)	**.044**	.087
STAXI Temperament	Parents	3.36 (1,44)	.074	.071
	Deployment	0.03 (1,44)	.856	.001
	Parents × Deployment	5.83 (1,44)	**.020**	.117
STAXI Anger Reaction	Parents	0.28 (1,44)	.600	.006
	Deployment	0.00 (1,44)	.988	.000
	Parents × Deployment	4.59 (1,44)	**.038**	.094

N = 49, Significant effects (*p* <.05) are shown in bold type.

### 3.3 Temperament and anger in relation to pre-deployment training and deployment status

The psychological outcomes were examined in more detail using two-factor analyses of variance, which considered both pre-deployment training (training versus no training) and deployment exposure (deployed versus non-deployed). The results of these analyses are presented in **Table 3**. A significant main effect of pre-deployment training was observed for temperament, *F (1*,40) = 9.26, *p* = .004, indicating lower temperament scores among firefighters who had received preparatory training. Similarly, a significant main effect of training was found for anger expression-out, *F (1*,41) = 11.99, *p* = .001, with lower anger expression among trained firefighters. In addition, significant interaction effects between training and deployment exposure were observed for temperament, *F (1*,40) = 5.55, *p* = .023, and anger expression-out, *F (1*,41) = 4.31, *p* = .044. No significant effects were found for anger reaction, and deployment exposure alone did not show independent effects across outcomes.

**Table 3:** Two-factor ANOVAs examining the effects of pre-deployment training and deployment exposure on anger-related outcomes.

Outcome variable	Effect	*F* (df1, df2)	*p*	*η_p_²*
STAXI Temperament	Training	9.26 (1,40)	**.004**	.188
	Deployment	0.96 (1,40)	.334	.023
	Training × Deployment	5.55 (1,40)	**.023**	.122
STAXI Anger Reaction	Training	3.68 (1,40)	.062	.084
	Deployment	0.65 (1,40)	.426	.016
	Training × Deployment	1.14 (1,40)	.293	.028
STAX Anger Ex Out	Training	11.99 (1,41)	**.001**	.226
	Deployment	1.15 (1,41)	.289	.027
	Training × Deployment	4.31 (1,41)	**.044**	.095

N = 47, Significant effects (*p* <.05) are shown in bold type.

## 4 Discussion

The present findings suggest that psychological outcomes following a child-related critical incident, such as the fatal traffic accident examined in the present study, may be more strongly associated with parental status than with deployment exposure. Firefighters with children reported lower environmental quality of life, irrespective of deployment status. However, anger-related variables exhibited interaction effects, suggesting that emotional responses varied depending on the combination of parental status and deployment exposure. Unlike our initial hypothesis, deployment exposure alone did not emerge as a significant predictor of psychological outcomes. This finding suggests that subjective personal relevance may be more significant than objective exposure in shaping post-incident responses. Parenthood may increase identification with child victims, and thus increase emotional salience, even in the absence of direct exposure. These results extend the scope of exposure-based models of occupational trauma by underscoring the significance of personal role salience. Therefore, psychological support strategies may benefit from considering parental status in addition to operational exposure.

Environmental quality of life is indicative of subjective appraisal rather than objective environmental conditions. In the context of emergency operations, firefighters are required to direct their attention towards potential sources of danger. As posited by stress appraisal models, situational cues have been shown to be evaluated as more threatening when individuals are under pressure, particularly when their attentional focus is concentrated on danger-related stimuli (33). In this context, when children are involved, personal identification processes may intensify emotional involvement, especially among firefighters who are parents. Fatal traffic accidents are well-established stressors for emergency personnel (34), especially when children are among the fatalities (35). Conversely, earlier research has indicated that comparable incidents lacking child fatalities were not associated with an elevated burden among firefighter parents (36). Taken together, these findings indicate that parental status may be associated with heightened subjective appraisal of environmental threat and reduced quality of life following child-related critical incidents.

Furthermore, we were able to show the importance of targeted training in preparation for deployment to cope with psychological burden. Firefighters who had received pre-deployment training reported lower levels of temperament and anger expression, suggesting that structured preparation may help mitigate emotional reactions during critical incidents. Training and operation simulations can help to better prepare rescue workers for major incidents and strengthen their psychological resilience, as shown in similar studies (37, 38). These findings emphasize the potential value of structured preparatory training in high-risk professions.

The present study did not identify any cases of PTSD in the investigated sample. Although a prevalence of up to 10% has been reported among emergency personnel, it is important to note that this represents an upper estimate and may not apply equally to all types of incidents. The absence of PTSD cases in the sample may be explained by several factors, including the limited scope of the traffic accident, the absence of a direct risk of death to the firefighters, and the fact that such incidents are generally considered part of routine occupational exposure. Pahnke et al. also identified comparable low levels of PTSD in their study of first responder samples (36). Low PTSD rates of 1.3% were also reported after a terrorist attack in Madrid (39). Furthermore, the implementation of effective psychological support structures may have served to mitigate the development of symptoms.

The present results underline the need for further research on the psychological stress of emergency services. Future studies should systematically investigate whether, and to what extent, the type of operation and a possible personal impact, for example through a connection to the victim, influence the experience of stress.

A limitation of our study is, that the data were collected by using questionnaires based on the subjective self-assessment of the emergency services. As a result, possible distortions due to conscious or unconscious misjudgments cannot be ruled out. Other important factors that can have an influence on mental health (e.g., genetic predisposition or social environment) are not recorded. Furthermore, no data on the mental health of the emergency services before the event were collected. If, for example, a participant was already suffering from a mental illness before the assignment, a direct connection between the assignment and the results can only be established to a limited extent. During the evaluation, it was noticed that not all participants completed all parts of the questionnaire, which can affect the completeness of the data. Another limitation is the small number of cases. With only 49 participants in total, statements about the entire Berlin Fire Department with its approximately 4,500 employees should be made with caution (40). It should also be noted that the 4,500 employees do not consist exclusively of emergency personnel. A significant proportion of the workforce is employed in back-office functions (e.g., administration) and is not directly exposed. Therefore, a differentiated approach is also necessary within the Berlin Fire Department. Furthermore, only 19 of the firefighters involved in the operation could be included in the study. Therefore, no generalized statement can be made about the operation. As previously stated in the results section, except for two female firefighters, only male firefighters participated in the survey. The results can therefore not be evaluated across genders. However, since firefighters in Germany are still predominantly male, the low number of female participants is currently representative of the circumstances (41). Further surveys on the second (12 months after the event) and third measurement time (24 months after the event) are still pending. These data could be used to provide more detailed information on the course of the results.

In summary, the present study demonstrates that parental status may influence psychological outcomes following child-related critical incidents in firefighters. Firefighters with children reported lower environmental and physical quality of life, while pre-deployment training was associated with lower levels of anger-related responses. Deployment exposure itself did not emerge as an independent predictor of psychological outcomes. These findings suggest that individual life circumstances and prior preparation may shape psychological responses to critical events and should be considered in the development of tailored preventive and support strategies in high-risk professions.

## Data Availability

The datasets presented in this article are not readily available because The data collected for this study is not publicly available due to privacy and ethical restrictions. Requests to access the datasets should be directed to Ulrich Wesemann, uw@ptzbw.org.
